# Ring-opening of non-activated aziridines with [^11^C]CO_2_*via* novel ionic liquids†

**DOI:** 10.1039/d2ra03966d

**Published:** 2022-08-02

**Authors:** Anton Lindberg, Neil Vasdev

**Affiliations:** Azrieli Centre for Neuro-Radiochemistry, Brain Health Imaging Centre, Centre for Addiction and Mental Health Toronto ON M5T 1R8 Canada anton.lindberg@camh.ca; Department of Psychiatry, University of Toronto Toronto ON M5T 1R8 Canada neil.vasdev@utoronto.ca

## Abstract

Novel ionic liquids based on DBU and DBN halide salts were developed as a catalytic system for ring-opening of non-activated aziridines with [^11^C]CO_2_. The ability of ionic liquids to activate aziridines represents a simple methodology for the synthesis of ^11^C-carbamates and can be extended for CO_2_-fixation in organic and radiochemistry.

## Abstract 2

Novel ionic liquids based on DBU and DBN halide salts were developed as a catalytic system for ring-opening of non-activated aziridines with [^11^C]CO_2_.

## Graphical abstract

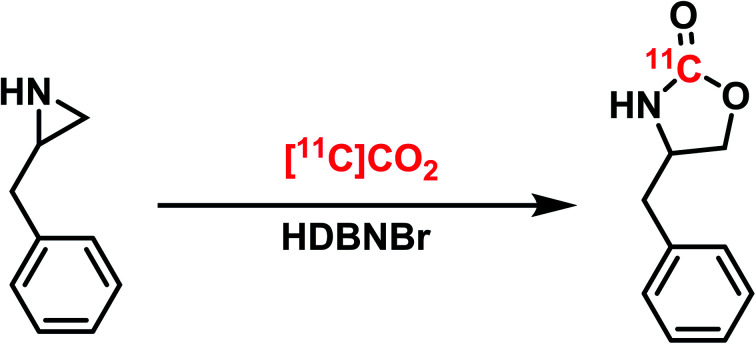

## Introduction

Positron emission tomography (PET) is a dynamic functional imaging technique used to study biochemical interactions *in vivo* with extensive applications in clinical trials and drug development. PET utilizes positron emitting radionuclides, frequently, carbon-11 (^11^C, *t*_1/2_ = 20.4 min) and fluorine-18 (^18^F, *t*_1/2_ = 109.7 min), which are incorporated into a biologically active molecule (radiotracers) that are injected *in vivo* and used to generate a tomographic image.^1^ Due to the relatively short half-life of ^11^C, radiochemical reactions with this radionuclide must be fast and efficient. Cyclotron-produced ^11^C is generated in target by the ^14^N(p,α)^11^C nuclear reaction as either [^11^C]CO_2_ or [^11^C]CH_4_ for conversion to [^11^C]CH_3_I or [^11^C]CH_3_OTf and applied for ^11^C-methylation reactions. More recently ^11^C-carbonylation reactions are being applied for clinical PET research studies, where [^11^C]CO_2_ is used directly or converted to [^11^C]CO, [^11^C]COCl_2_ or [^11^C]HCN.^2^ While recent developments of [^11^C]CO reactions have been made,^3^ its use is still limited by the poor solubility in organic solvents, low reactivity and often need for pressurized reactors. The conversion of [^11^C]CO_2_ to [^11^C]CO is relatively time consuming and inefficient. [^11^C]COCl_2_ is more reactive than [^11^C]CO for ^11^C-carbonylation reactions, however its production requires specialized equipment and use of chlorine gas.^4^^11^C-carbonylation can also be achieved using [^11^C]HCN, but is limited primarily to ^11^C-carboxylic acids and primary ^11^C-amides.^5^ Using [^11^C]CO_2_ directly for ^11^C-carbonylation removes the need for converting the cyclotron produced [^11^C]CO_2_ into other C_1_ building blocks and can be used directly for the syntheses of a wide range of ^11^C-carbonyl functionalities.^6^

**Fig. 1:**
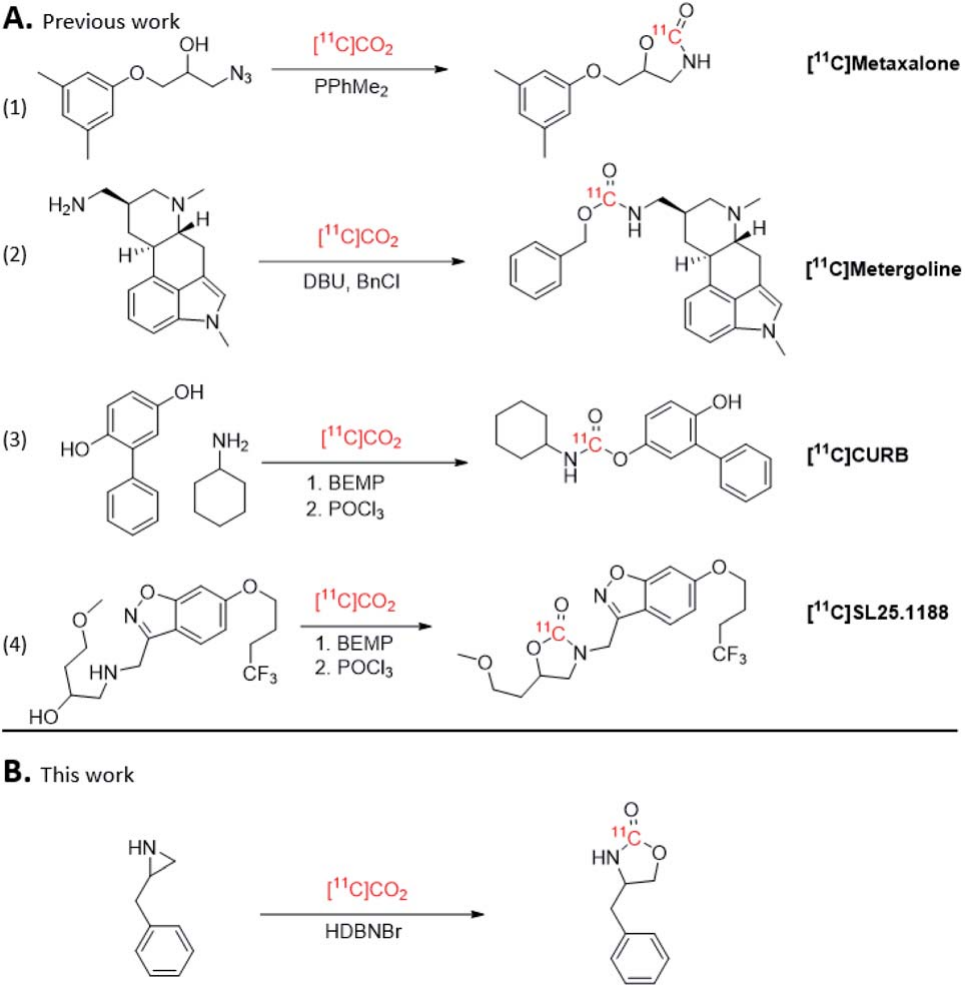
(A) Previous syntheses of ^11^C-carbamates using [^11^C]CO_2_. (B) This work.

[^11^C]CO_2_ fixation reactions have gained widespread use for ^11^C-carbonylation of PET radiotracers,^7^ and are often achieved by trapping [^11^C]CO_2_ directly from the cyclotron in a bulky organic base, such as 2-*tert*-butylimino-2-diethylamino-1,3-dimethylperhydro-1,3,2-diazaphosphorine (BEMP), 1,8-diazabicyclo[5.4.0]undec-7-ene (DBU) or 1,5-diazabicyclo[4.3.0]non-5-ene (DBN) under ambient conditions. This method of [^11^C]CO_2_ fixation been successfully utilized to generate ^11^C-carbamates in high radiochemical yields (Fig. 1A),^8–11^ including [^11^C]CURB and [^11^C]SL25.1188 that were developed and translated for clinical PET research by our laboratories (Fig. 1A entries 3 and 4).^12,13^

The use of ionic liquids (ILs)^14–19^ as a catalytic system for trapping of CO_2_ from the atmosphere has been reported in environmental chemistry^20^ and organic synthesis. For example, IL-catalysed CO_2_ insertion by ring-opening of aziridines or epoxides^21^ have been explored to form cyclic carbamates and carbonates, respectively.^22–26^ Inserting CO_2_ into epoxides to form cyclic carbonates, such as dioxolan-2-ones, have been shown to be an efficient way to bind atmospheric CO_2_.^26^ However, the resulting cyclic carbonates are prone to ring-open in aqueous conditions.^27^ However, CO_2_ insertion into aziridines forms oxazolidine-2-ones, which are a common motif in pharmaceuticals as well as [^11^C]SL2511.88 (Fig. 1A), and has been achieved by ring-opening aziridines with CO_2_ using metal or organic halides.^22^ Relevant to [^11^C]CO_2_ fixation, ILs based on halide salts derived from DBU have been reported for ring-opening of epoxides with CO_2_.^28^ While ILs have been employed for ^18^F-radiochemistry,^29,30^ to our knowledge, ILs have not been explored in ^11^C- radiochemistry.

Ring-opening of activated aziridines and epoxides have been reported in PET radiochemistry using both ^11^C and ^18^F nucleophiles, namely, [^11^C]HCN and [^18^F]fluoride.^31–35^ In the present study, we describe the application of ILs, including novel DBU and DBN halide salts, to assess their ability to trap [^11^C]CO_2_ and ring-open aziridines to form cyclic [^11^C]carbamates (Fig. 1B).

## Methods

No-carrier added [^11^C]CO_2_ was produced on a MC-17 cyclotron (Scanditronix, Sweden) using the ^14^N(p,α)^11^C nuclear reaction in a pressurized gas target contain a nitrogen : oxygen mixture (99.5 : 0.5). The radiosynthesis was performed using a TracerMaker™ carbon-11 synthesis platform (Scansys Laboratorieteknik, Denmark). Analytical HPLC was performed on a 1260 Infinity II HPLC system (Agilent, CA) with a M177 γ-radiation detector (Ludlum Instruments, TX) connected in series after the UV detector. Radiochemistry was performed in lead-shielded hot cells in a laboratory designed to handle radioactive material. The work was performed in accordance with the radiation protection guidelines and regulations from the Canadian Nuclear Safety Commission and internal safety policies and procedures.

### Chemistry

Synthesis of HDBU and HDBN halide salts were performed according to a previously reported method.^28^ DBU/DBN (20 mmol) and ammonium halide (20 mmol) was taken up in methanol (50 mL) and refluxed overnight. Volatiles were removed *in vacuo* to give the desired IL as a solid (see ESI† for details).

### Radiochemistry

[^11^C]CO_2_ was first trapped on a HayeSep D column (700 mg) cooled to −180 °C using liquid nitrogen on a TracerMaker™ synthesis platform. Upon heating the HayeSep D column, [^11^C]CO_2_ was released and carried by helium gas to a reaction vessel pre-charged with IL (10–20 mg), aziridine (5 mg) and NMP (200 μL), heated at 85 °C for 15 min under argon atmosphere prior to addition of [^11^C]CO_2_. After maximum radioactivity in the reaction vessel was reached, the transfer lines were removed and the reaction was heated for 5 min. An ascarite trap was connected to the outlet line from the vial during transfer from the HayeSep D column to trap unreacted [^11^C]CO_2_. After completion of the reaction, the gas in the headspace of the reaction vessel was vented through the same ascarite trap. [^11^C]CO_2_ trapping efficiency (TE) was calculated as the amount of activity in the reaction mixture after venting compared to the amount on the ascarite trap. Radiochemical conversion (RCC) was subsequently measured by radio-HPLC from an aliquot of the crude reaction mixture and calculated as the amount of desired product compared to untrapped [^11^C]CO_2_ and any byproducts in the radio-HPLC chromatogram.

## Results and discussion

Ring-opening of benzylaziridine with [^11^C]CO_2_ to produce [^11^C]benzyloxazolidin-2-one ([^11^C]1) using tetrabutylammonium bromide (TBABr) and NMP was used as a starting point for reaction optimization (Table 1). Reactions in the absence of an activation step were carried out at 130 °C for 5 min, and only reached a maximum TE of 24% to produce [^11^C]1 with a RCC of 24%. Introduction of an activation step prior to [^11^C]CO_2_ addition proved to be effective at increasing the TE. By heating benzylaziridine and the TBABr mixture to 85 °C for 15 min prior to adding [^11^C]CO_2_, the TE increased to 45% with a higher RCC of 41% (Table 1, entry 1). Reducing the reaction time to 1 min lowered the overall RCC to 37% and decreasing the reaction temperature to 70 °C or ambient temperature lowered the RCC to 29% and 11%, respectively (entries 2–4). 1-Butyl-3-methylimidazolium bromide (BMIMBr), a commercially available quaternary ammonium IL that has been applied for ring-opening of epoxides with CO_2,_^26^ resulted in a lower TE and RCC (28% and 27%, respectively; entry 5). Lithium bromide has also been used in ring-opening reactions with CO_2_ on epoxides and aziridines, however, the poor solubility of LiBr in NMP is attributed to the low overall RCC (3%) in this model reaction (entry 6), despite having similarly effective TE as achieved using TBABr.

**Table 1:** TEs and RCCs for the radiosynthesis of [^11^C]benzyloxazolidin-2-one from benzylaziridine and [^11^C]CO_2_ using ILs^a^

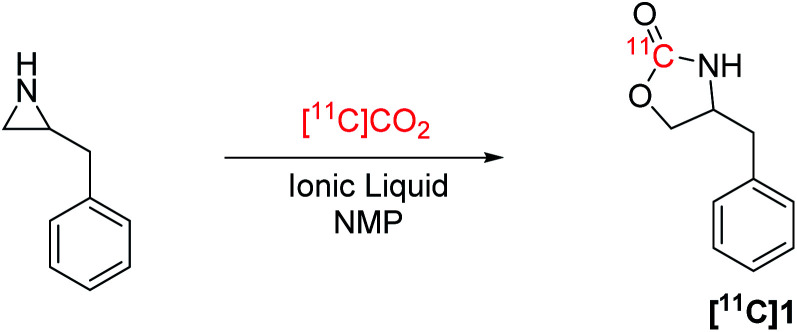				
Entry	Ionic liquid/salt	Amount	TE	RCC
1	TBABr	10 mg	45%	41%
2	TBABr*	10 mg	38%	37%
3	TBABr**	10 mg	30%	29%
4	TBABr***	10 mg	13%	11%
5	BMIMBr	10 mg	28%	27%
6	LiBr	10 mg	45%	3%
7	TBABr (BEMP)	10 mg	99%	—
8	LiBr (DBU)	10 mg	99%	—
9	TBABr (DBU)	10 mg	98%	8%
10	HDBUI	10 mg	53%	50%
11	HDBUBr	10 mg	69%	67%
12	HDBUCl	10 mg	59%	57%
13	HDBNI	10 mg	55%	54%
14	HDBNI	20 mg	64%	63%
15	HDBNCl	20 mg	68%	64%
**16**	**HDBNBr**	**20 mg**	**78%**	**77%**

a General conditions, with IL (10–20 mg), aziridine (5 mg) and NMP (200 μL), activated at 85 °C for 15 min under argon. Reaction time 5 min at 130 °C. * 1 min reaction time, ** 70 °C reaction temperature, *** ambient reaction temperature.

Traditional bases used in [^11^C]CO_2_ fixation, namely BEMP or DBU, increased the TE to 99% but were not suitable for forming the ring-opened product, [^11^C]1 (entries 7–9). It is postulated that the fixating agents bind [^11^C]CO_2_ without coordinating to the aziridine and therefore restricts the [^11^C]CO_2_ insertion reaction, whilst allowing for high TE. HDBUI, which has been reported as an effective IL for opening epoxides with CO_2_,^28^ proved to also be effective for ring-opening benzylaziridine with [^11^C]CO_2_ and showed TE of 50% with RCC of 46% (entry 10). Analogues of HDBUI were subsequently investigated. Changing the anion from iodide to bromide gave TE of 69% and RCC of 67% (entry 11). However, using chloride as the anion gave TE of 59% and RCC of 57% (entry 12). Use of DBN instead of DBU as the cation and iodide as the anion gave TE of 55% and RCC of 54% (entry 13). Increasing the amount of IL used from 10 mg to 20 mg resulted in 64% TE and 63% overall RCY using HDBNI (entry 14). The chloride and bromide salts of DBN were synthesized and resulted in improved TE of 68% and RCC of 64% using HDBNCl (entry 15). The optimized synthesis of [^11^C]1 was achieved using HDNBr and resulted in a TE of 78% with RCC of 77% (entry 16).

Only one product resulted in all experiments despite the potential for benzyl aziridine to ring-open *via* two different pathways to form either [^11^C]4-benzyloxazolidin-2-one or [^11^C]5-benzyloxazolidin-2-one. The absolute enantiomeric configuration of the product was confirmed by performing the reaction with CO_2_ instead of [^11^C]CO_2_. The reaction yielded only one product which co-eluted with the radiochemical product by HPLC analysis (see ESI† for details). ^1^H- and ^13^C-NMR spectroscopic analysis comparing the synthesized product with a sample of authentic 5-benzyloxazolidin-2-one confirmed that both the radiochemical and chemical product was 4-benzyloxazolidin-2-one.

With the optimized conditions (Table 1, entry 16) using HDBNBr and 15 min activation at 85 °C prior to addition of [^11^C]CO_2_ followed by 5 min reaction at 130 °C, the substrate scope of this reaction was evaluated with commercially available aziridines (Table 2). *N*-Tosylated aziridines (activated aziridines) did not yield a ^11^C-carbamate product. However, the same reaction conditions applied to non-activated *N*-protonated and *N*-alkylated azirdines resulted in ^11^C-carbamates as the products. Four aziridines were successfully labelled with [^11^C]CO_2_ using HDBNBr as IL. Only one radiochemical product was detected in all reactions. Performing the reaction with the spirocyclic precursor 1-azaspiro[2.5]octane (Table 2, entry 1) resulted in 50% TE and 5% RCC of [^11^C]oxa-azaspirodecan-2-one ([^11^C]2). 1-Methyl-2-phenylaziridine was converted to [^11^C]phenyloxazolidin-2-one ([^11^C]3) with 55% TE and 46% RCC. 2,2-Dimethylaziridine produced [^11^C]dimethyloxazolidin-2-one ([^11^C]4) with 74% TE and 63% RCC. 1-Azabicyclo[3.1.0]hexane gave [^11^C]tetrahydro-1*H*,3*H*-pyrrolo[1,1-*c*]oxazol-3-one ([^11^C]5) with 95% TE and RCC. The relatively low RCC for [^11^C]2 are attributed to the steric hindrance by the conformation of the intermediate forming a stable “chair” configuration thus making the formation of a five-membered oxazolidinone unfavourable (Table 2, entry 1). A methylated aziridine was tolerated in this method (Table 2, entry 2). Dimethylaziridne showed TE and RCC comparable to benzylaziridine (Table 2, entry 3). The TE and RCC for formation of the bicyclic [^11^C]5 were the highest seen in this investigation and could be explained by the reaction mechanism which goes through an intermediate that releases the structural tension of the precursor and favours the formation of the bicyclic product [^11^C]5 (Table 2, entry 4).

**Table 2:** TEs and RCCs for radiosynthesis of [^11^C]2–[^11^C]6 using HDBNBr. Absolute configuration of the ring-opened product was not determined. All values are averages of 2 experiments

Entry	Aziridine	TE	RCC	Product
1	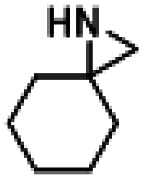	50%	5%	[^11^C]2
2	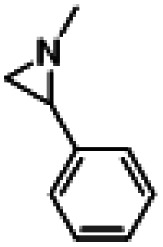	55%	46%	[^11^C]3
3	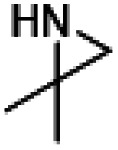	74%	63%	[^11^C]4
4	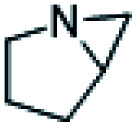	95%	95%	[^11^C]5
5	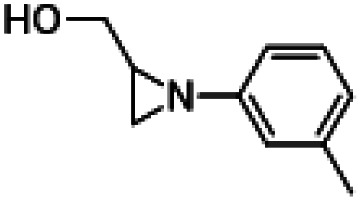	76%	1%	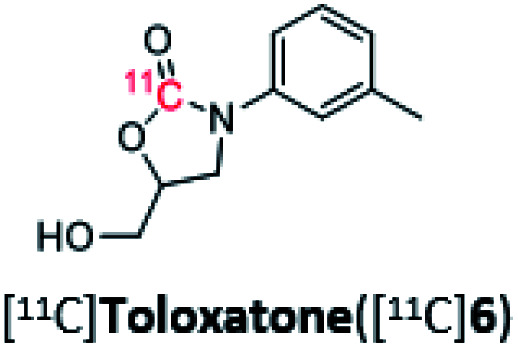

Toloxatone is a monoamine oxidase A inhibitor that has been synthesized from (1-(*m*-tolyl)aziridin-2-yl)methanol in an analogous aziridine ring-opening reaction, albeit using CO_2_ and metal catalysis.^36^ The radiosynthesis of [^11^C]toloxotone in our study ([^11^C]6; Table 2, entry 5) resulted with a TE of 76% and three radiochemical products were detected in the reaction mixture by radio-HPLC (Fig. S6†). The desired product ([^11^C]6) corresponded to *ca.* 1% RCC. Further optimization to improve the RCC were attempted but unsuccessful, and may be due to the alternative enantiomeric ring-opening reaction occurring. Due to the low RCC, isolation of [^11^C]6 was not possible and therefore the molar activity was not determined.

## Conclusions

Ionic liquids are demonstrated as an effective catalytic system for the radiosynthesis of ^11^C-carbamates by ring-opening of non-activated aziridines with [^11^C]CO_2_. Novels ILs based on DBN were synthesized herein and used to efficiently synthesize [^11^C]4-benzyloxazolidinone. This methodology was successfully used to ring-open four other non-activated aziridines to form ^11^C-carbamates. ILs are under exploration for further use with aziridines, epoxides in organic and radiochemistry.

## Author contributions

AL: conceptualization, synthesis, data analysis, wrote and edited the manuscript; NV: data analysis and edited the manuscript.

## Conflicts of interest

The authors declare no relevant conflicts of interest.

## Supplementary Material

RA-012-D2RA03966D-s001
