# Supplementary material for: Ring-opening of non-activated aziridines with [11C]CO2via novel ionic liquids
Source: RSC Adv. 2022 Aug 2;12(33):21417–21. doi: 10.1039/d2ra03966d (PMC9345297; doi:10.1039/d2ra03966d)
Supplement: RA-012-D2RA03966D-s001 [file RA-012-D2RA03966D-s001.pdf]

Electronic Supplementary Material (ESI) for RSC Advances.
This journal is © The Royal Society of Chemistry 2022

Supplemental information

Ring-opening of non-activated aziridines with [^{11}C]CO_2 *via* novel ionic liquids

Anton Lindberg[a*], and Neil Vasdev[ab*]

[a] *Azrieli Centre for Neuro-Radiochemistry, Brain Health Imaging Centre, Centre for Addiction and Mental Health, Toronto, ON, M5T 1R8, Canada.*
[b] *Department of Psychiatry, University of Toronto, Toronto, ON, M5T 1R8, Canada.*

1. **Radiochemistry**
   1.1 General procedure for [^{11}C]CO_2 chemistry **2**
   1.2 Radio-HPLC chromatograms of products [^{11}C]**1**-[^{11}C]**6** **2-4**
2. **Chemistry**
   Experimental procedures **5-11**
   Proposed mechanism for ring-opening of benzylaziridine with [^{11}C]CO_2 with HBNBr. **12**

General procedure for radiolabeling of [^{11}C]oxazolidin-2-ones. The reaction mixture was prepared in advance by adding aziridine (20 µmol) and Ionic liquid (10-20 mg) to a vial under argon atmosphere. N-Methyl-2-Pyrrolidone (NMP, 200 µL) was added and the vial was placed in the reactor on a TracerMaker^{TM} synthesis platform 15 min before end of bombardment and heated to 85 °C.

Cyclotron produced non-carrier added [^{11}C]CO_2 was trapped on a HayeSep D column (700 mg) cooled to -180 °C using liquid nitrogen on a TracerMaker^{TM} synthesis platform. The HayeSep D column was first slowly heated to ambient temperature and bubble through a closed reaction vial (4 mL) containing the prepared reaction mixture. The exhaust line from the reaction vial was connected to an ascarite (10 mg) trap. The reaction was then heated to 130 °C, 5 min before being cooled to 50 °C, and the headspace of the reaction vial was vented through the ascarite trap. An aliquot was taken from the reaction sample and analyzed by analytical HPLC (Alltima C18 5 µm, 250 x 4.5 mm, HiChrom) eluted with a gradient of MeCN: ammonium formate$_{aq}$ (15:85 to 85:15 v/v, 50mM) at 2 mL/min for 8 min.

Trapping efficiency (TE) was calculated as the fraction of radioactivity in the vial after venting compared to the total radioactivity in the vial and ascarite trap. Radiochemical conversion (RCC) was determined by radio-HPLC as the area under the curve for the product peak compared to total sample.

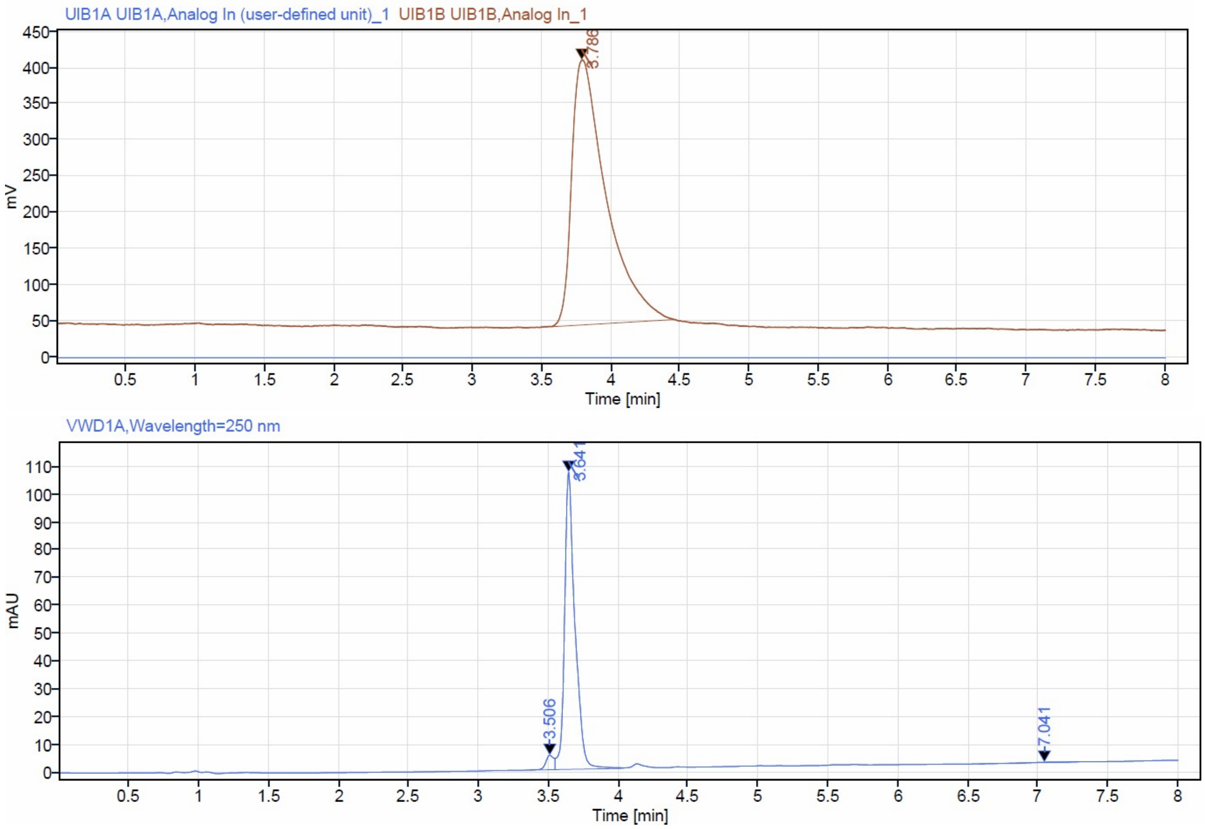

Figure S1. HPLC chromatogram of [^{11}C]**1** co injected with reference standard (t_R = 3.6- 4.4 min).

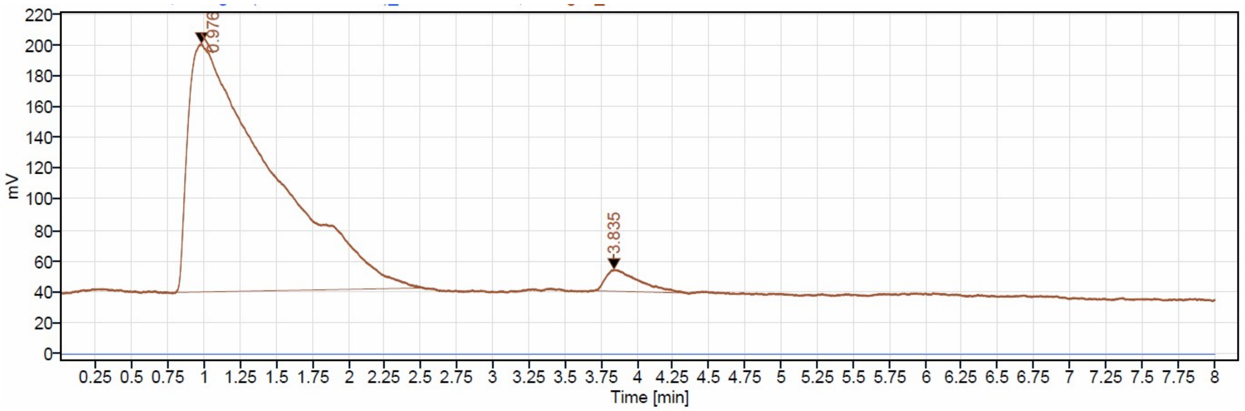

Figure S2. Radio-HPLC chromatogram of [^{11}C]**2** (t_R = 3.75- 4.25 min).

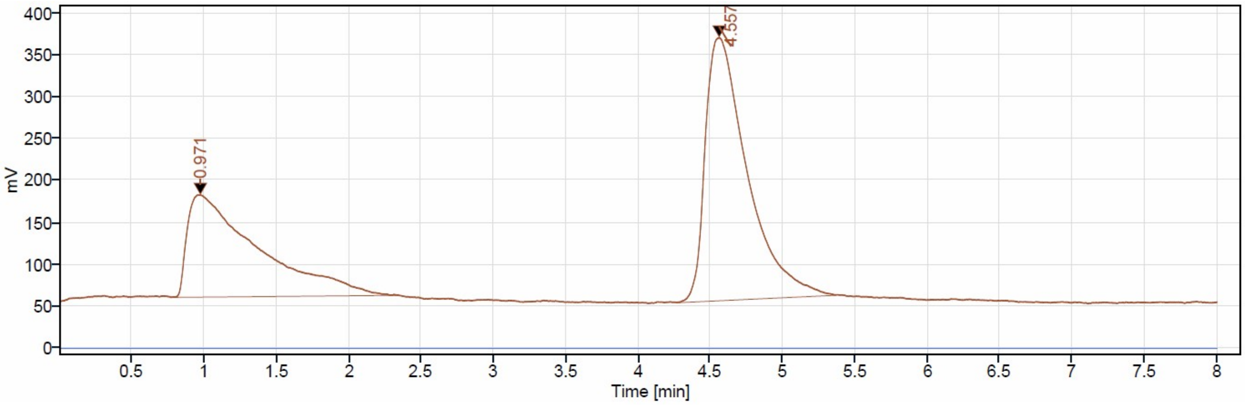

Figure S3. Radio-HPLC chromatogram of [^{11}C]**3** (t_R = 4.3- 5.3 min).

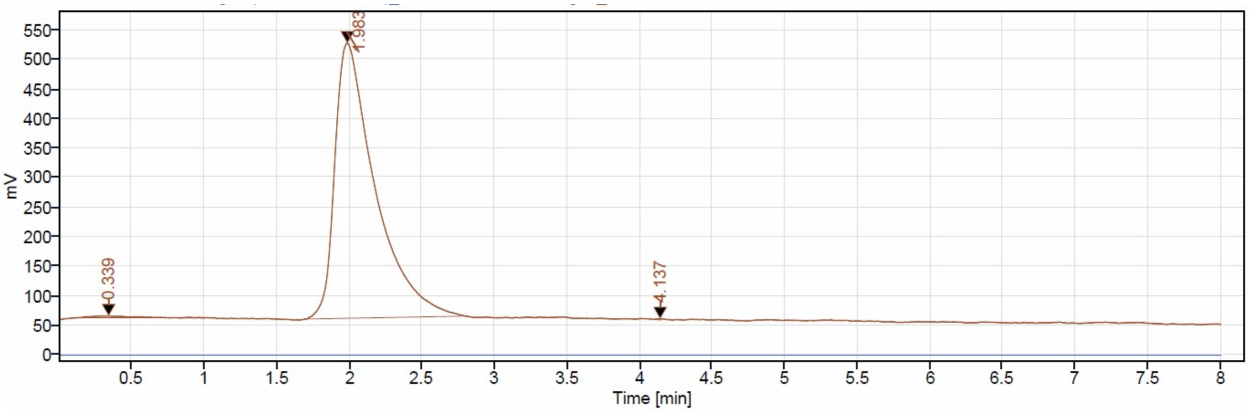

Figure S4. Radio-HPLC chromatogram of [^{11}C]**4** (t_R = 1.75- 2.75 min).

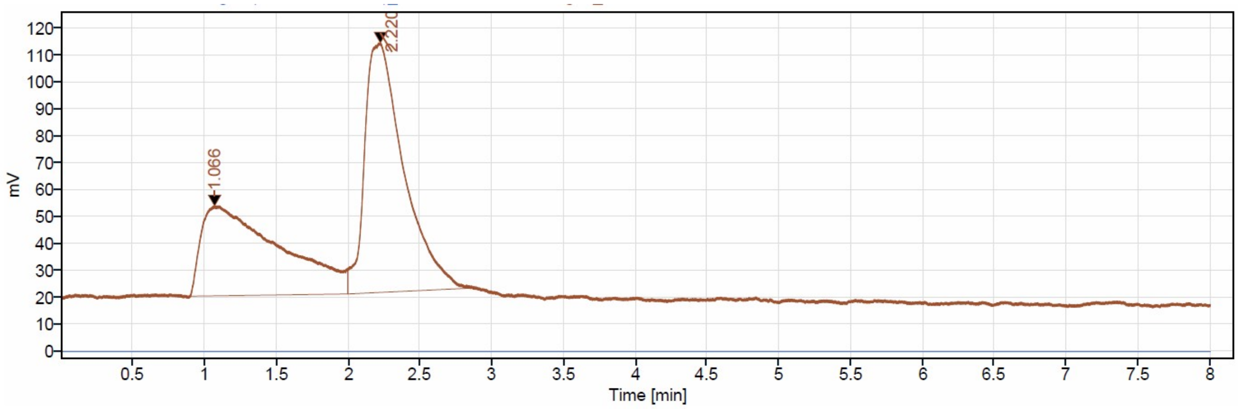

Figure S5. Radio-HPLC chromatogram of [^{11}C]**5** (t_R = 2.0- 2.75 min).

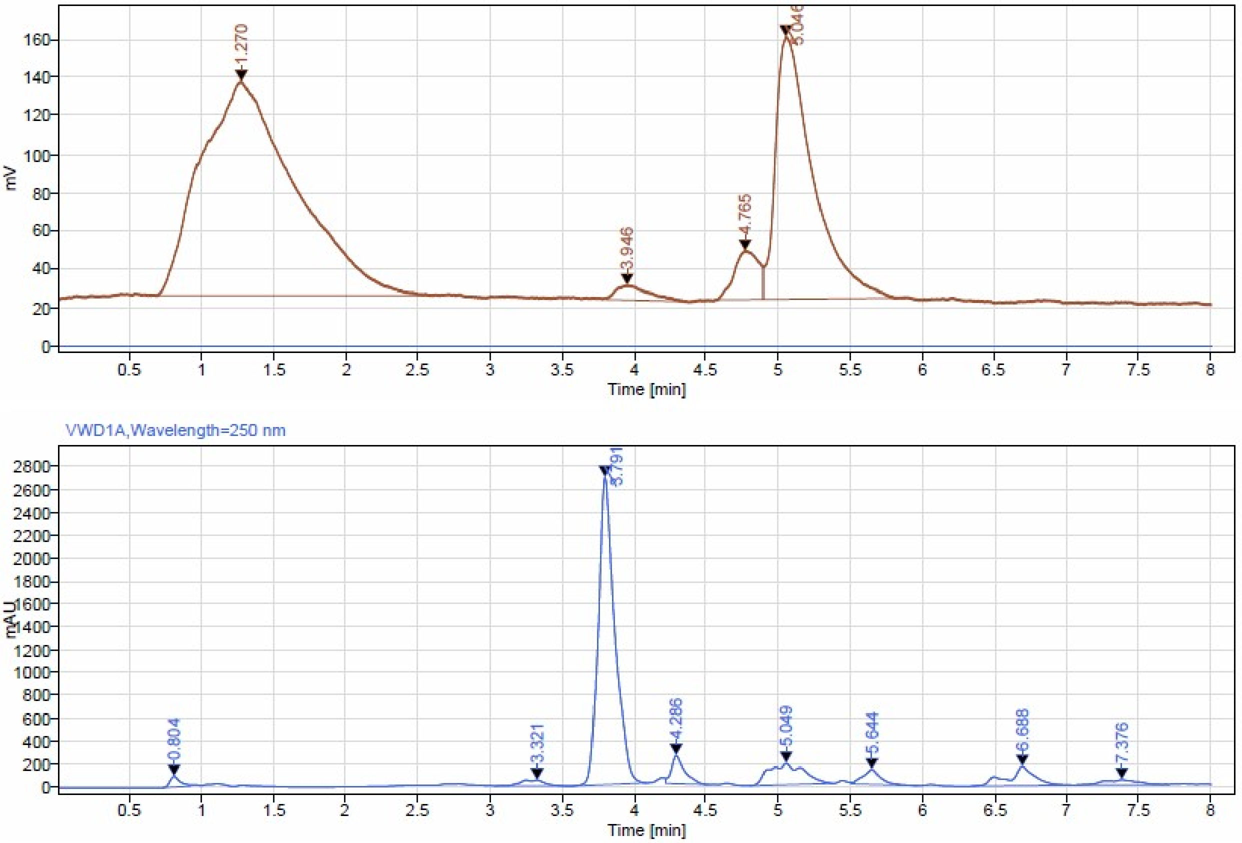

Figure S6. Radio and UV chromatograms of [^{11}C]toloxatone ([^{11}C]**6**)co-injected with authentic toloxatone standard

General procedure for synthesis of HDBN halide ionic liquids. Ammonium halide (20 mmol) and ammonium halide (20 mmol) was taken up in methanol (50 mL) and refluxed overnight. Volatiles were removed under *in vacuo* and hexane (3*50 mL) was added each time followed evaporation to form the desired salt as a solid.

*H-1,5-Diazabicyclo[4.3.0]non-5-ene Iodide (**HDBNI**)*. Quantitative yields. ^{1}H NMR (400 MHz, $CDCl_3$) δ 8.93 (s, 1H), 3.55 (t, 2H), 3.32 (t, 2H), 3.22 (t, 2H), 2.84 (t, 2H), 1.96 (p, 2H), 1.91 – 1.83 (m, 2H). ^{13}C NMR (101 MHz, $CDCl_3$) δ 164.09, 78.18, 77.86, 77.54, 53.79, 42.82, 37.78, 30.52, 18.83, 18.52.

*H-1,5-Diazabicyclo[4.3.0]non-5-ene Bromide (**HDBNBr**)*. Quantitative yields. ^{1}H NMR (400 MHz, $CDCl_3$) δ 9.43 (broad s, 1H), 3.63(t, 2H), 3.40 (t, 2H), 3.33 (t, 2H), 2.96 (t, 2H), 2.06 (p,2H), 1.95 (p, 2H). ^{13}C NMR (101 MHz, $CDCl_3$) δ 164.28, 53.57, 42.73, 37.9, 30.34, 18.87, 18.62.

*H-1,5-Diazabicyclo[4.3.0]non-5-ene Chloride (**HDBNCl**)*. Quantitative yields. ^{1}H NMR (400 MHz, $CDCl_3$) δ 10.76-10.34 (broad s, 1H), 3.38 (t, 2H), 3.15 (t, 2H), 3.06 (t, 2H), 2.66 (t, 2H), 1.80 9p, 2H), 1.68 (p, 2H). ^{13}C NMR (101 MHz, $CDCl_3$) δ 163.92, 53.20, 49.35, 42.40, 37.72, 29.9, 18.59, 18.41.

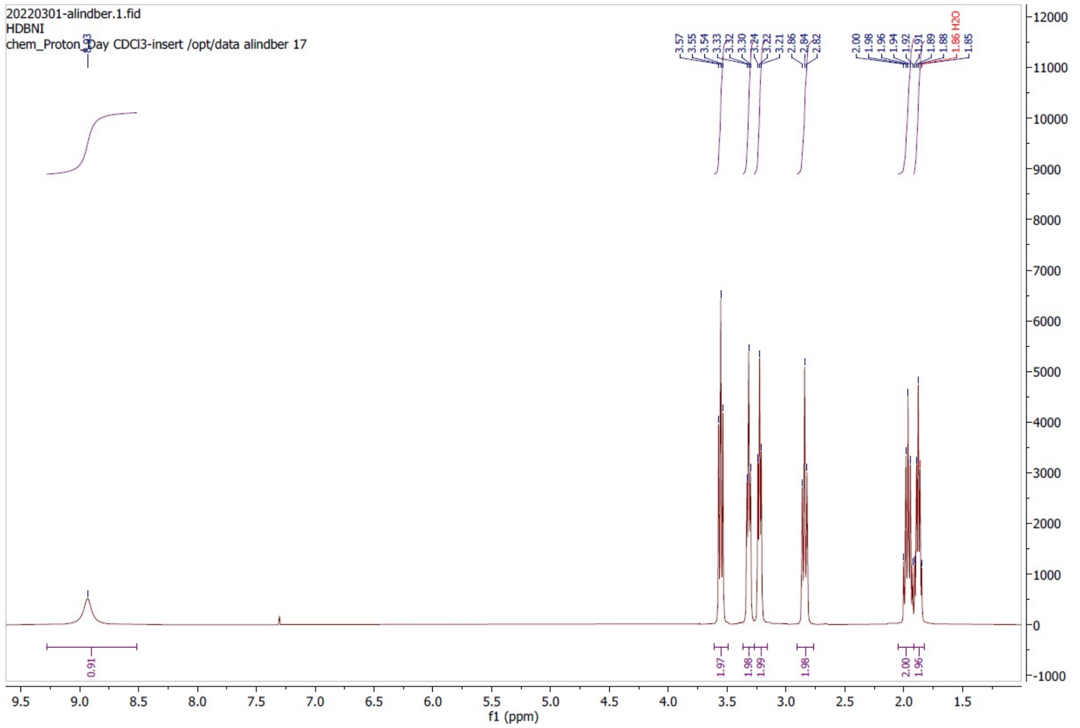

Figure S7. ^{1}H-NMR of HDBNI.

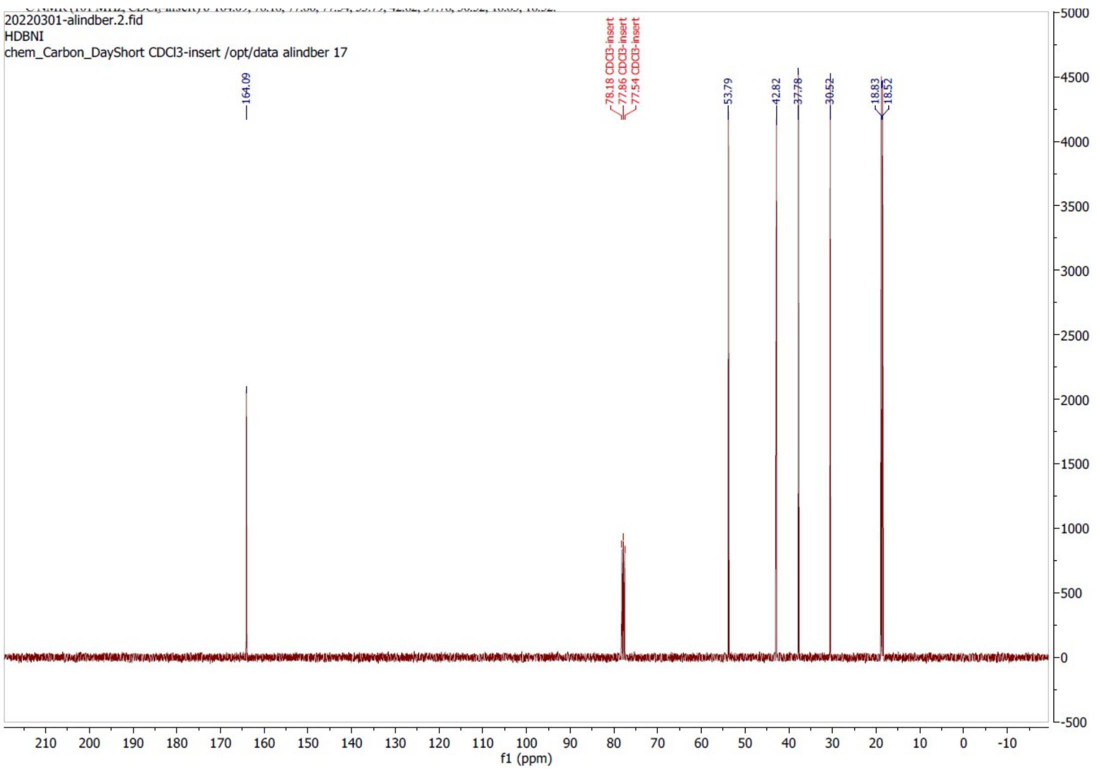

Figure S8. ^{13}C-NMR of HDBNI.

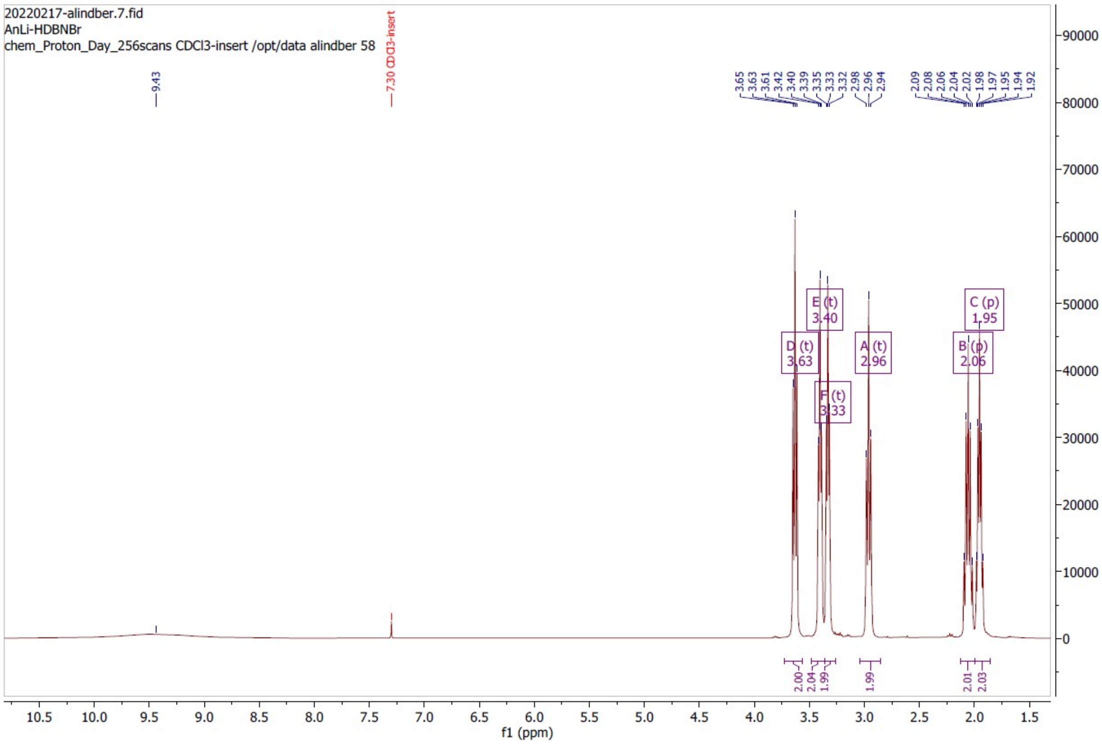

Figure S9. ^{1}H-NMR of HDBNBr.

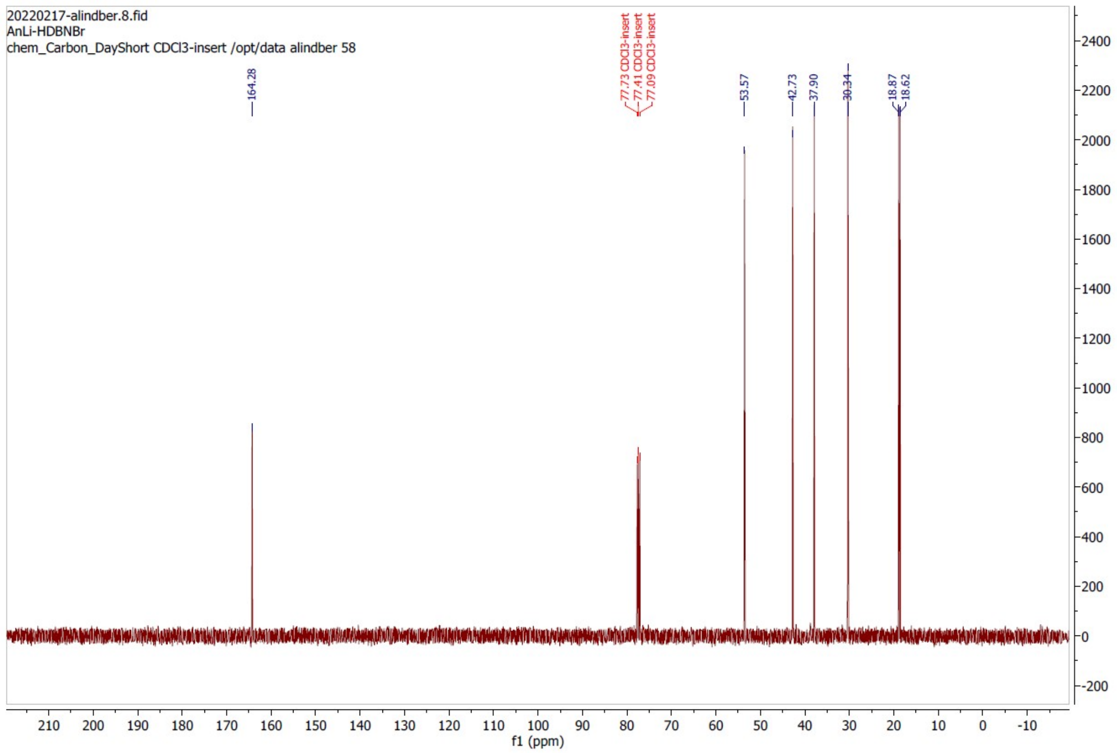

Figure S10. ^{13}C-NMR of HDBNBr.

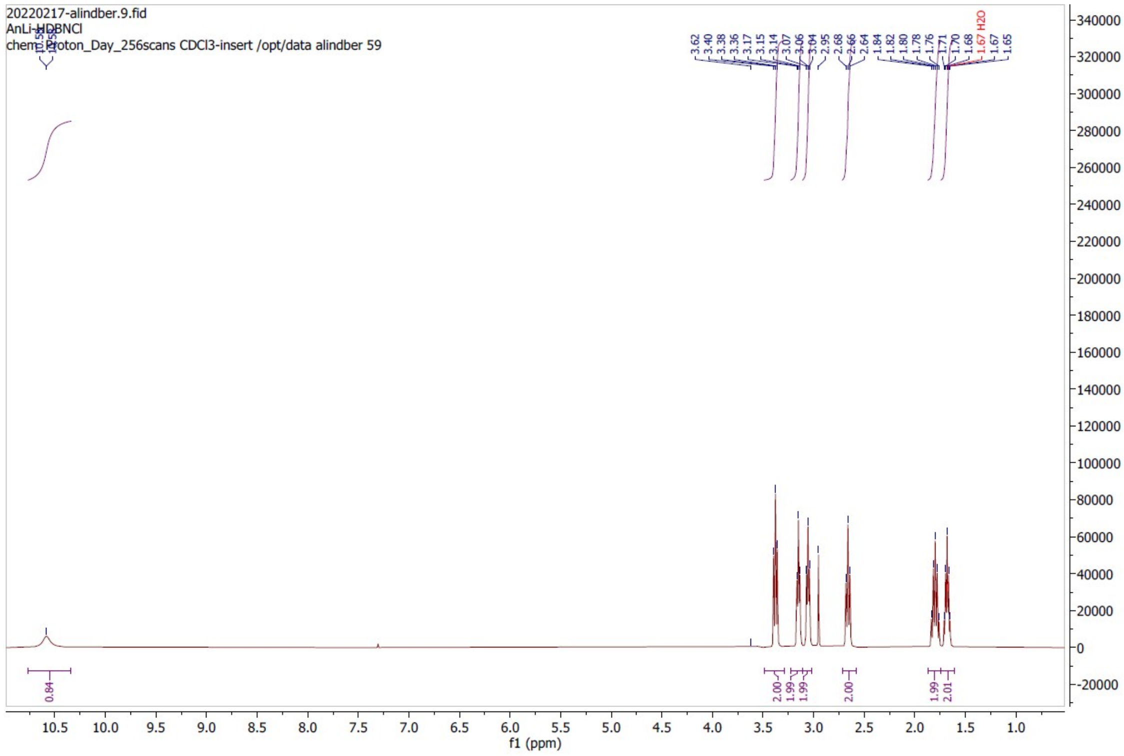

Figure S11. ^{1}H-NMR of HDBNCl.

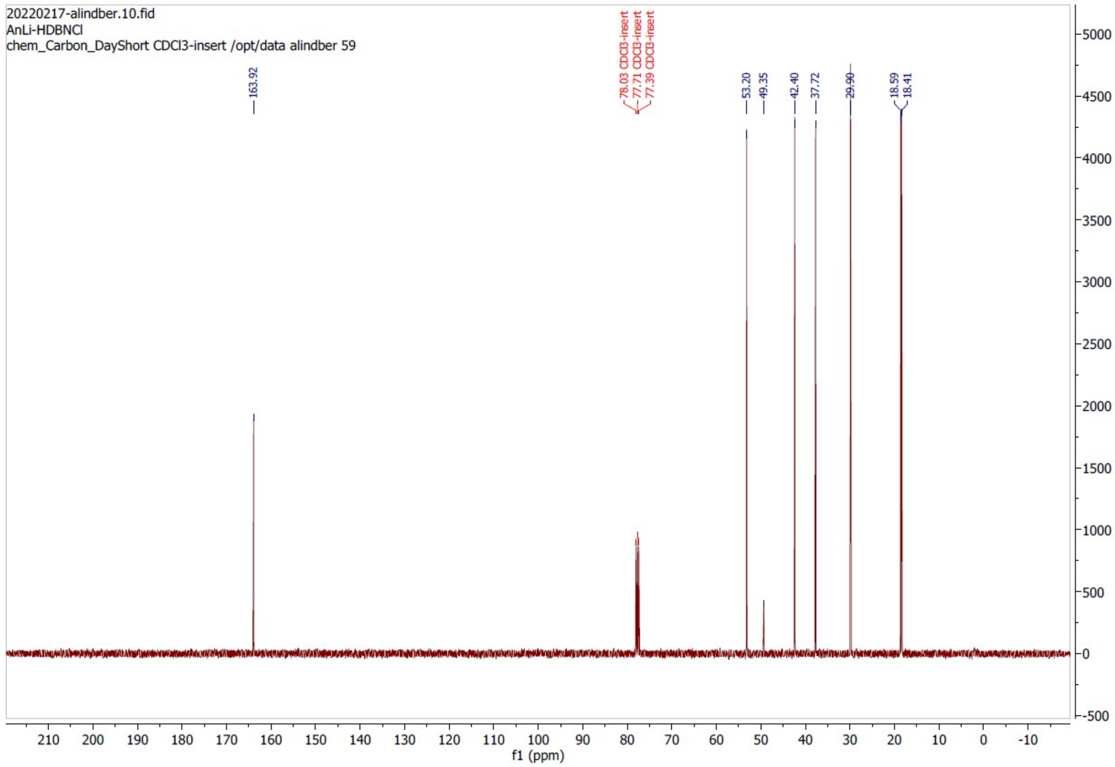

Figure S12. ^{13}C-NMR of HDBNCl.

*4-benzyloxazolidin-2-one (**1**).* Benzylaziridine (15 mg, 110 µmol) and HDBNBr (60 mg) in a capped vial under argon atmosphere. N-Methyl-2-Pyrrolidone (NMP, 600 µL) was added. The mixture was heated to 85°C for 15 min before CO_2 was bubbled through the solution for 5 min. The reaction was heated to 130 °C for 10 min. Water (30 mL) was added and was extracted with ethyl acetate (3*20 mL), organics were pooled and dried with $MgSO_4$, filtered and evaporated. Product was isolated by flash column chromatography using ethyl acetate (5-40 %) in heptane to give **1** (10 mg, 56 µmol, 50% yield). ^{1}H NMR (500 MHz, $CDCl_3$) δ (dd, *J* = 8.1, 6.6 Hz, 2H), 7.29 – 7.19 (m, 1H), 7.19 – 7.12 (m, 2H), 6.12 (s, 1H), 4.40 (t, 1H), 4.17-4.02 (m, 2H), 2.90 (dd, 1H), 2.86-2.77 (m,1H). ^{13}C NMR (500 MHz, $CDCl_3$) δ 159.62, 135.95, 129.05, 128.95, 127.19, 69.55, 53.77, 41.35. HRMS (DART+) *m/z* $[M+H]^+$ calculated for $C_{10}H_{12}NO_2$ 178.08626, found 178.08628

^{1}H NMR (500 MHz, cdcl$_3$) δ 7.32 (dd, J = 8.1, 6.6 Hz, 2H), 7.29 – 7.19 (m, 1H), 7.19 – 7.12 (m, 2H), 6.12 (s, 1H), 4.40 (t, J = 8.1 Hz, 1H), 4.16 – 4.03 (m, 2H), 2.90 (dd, J = 13.6, 6.8 Hz, 1H), 2.86 – 2.79 (m, 1H).

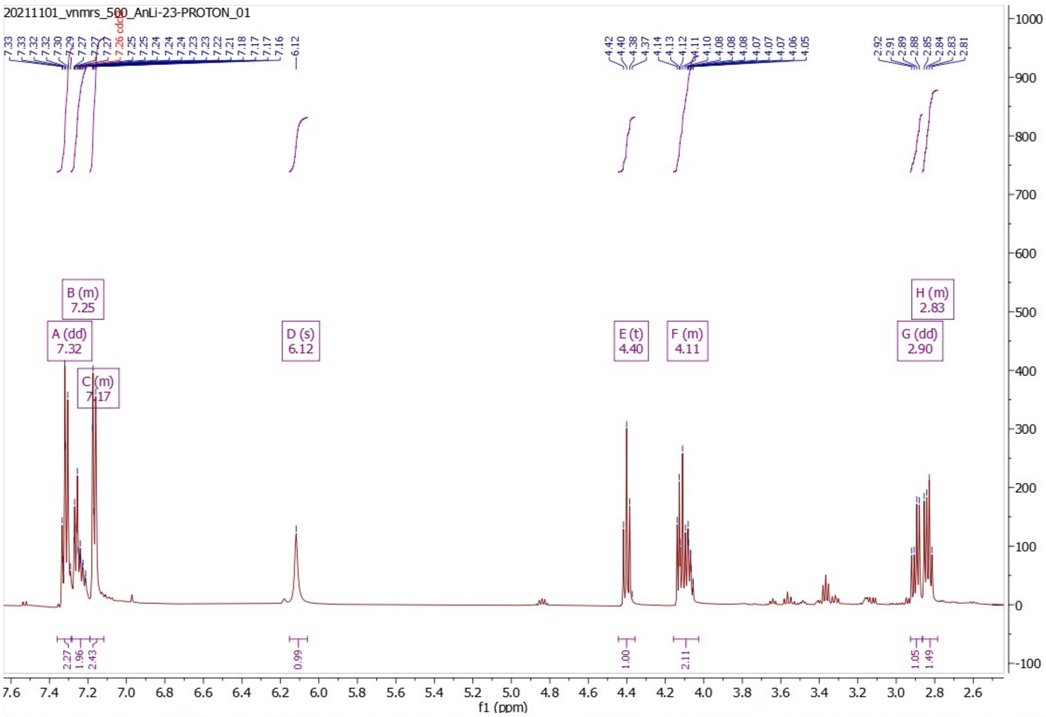

^{1}H NMR (500 MHz, cdcl$_3$) δ 7.32 (dd, J = 8.1, 6.6 Hz, 2H), 7.29 – 7.19 (m, 1H), 7.19 – 7.12 (m, 2H), 6.12 (s, 1H), 4.40 (t, J = 8.1 Hz, 1H), 4.16 – 4.03 (m, 2H), 2.90 (dd, J = 13.6, 6.8 Hz, 1H), 2.86 – 2.79 (m, 1H).

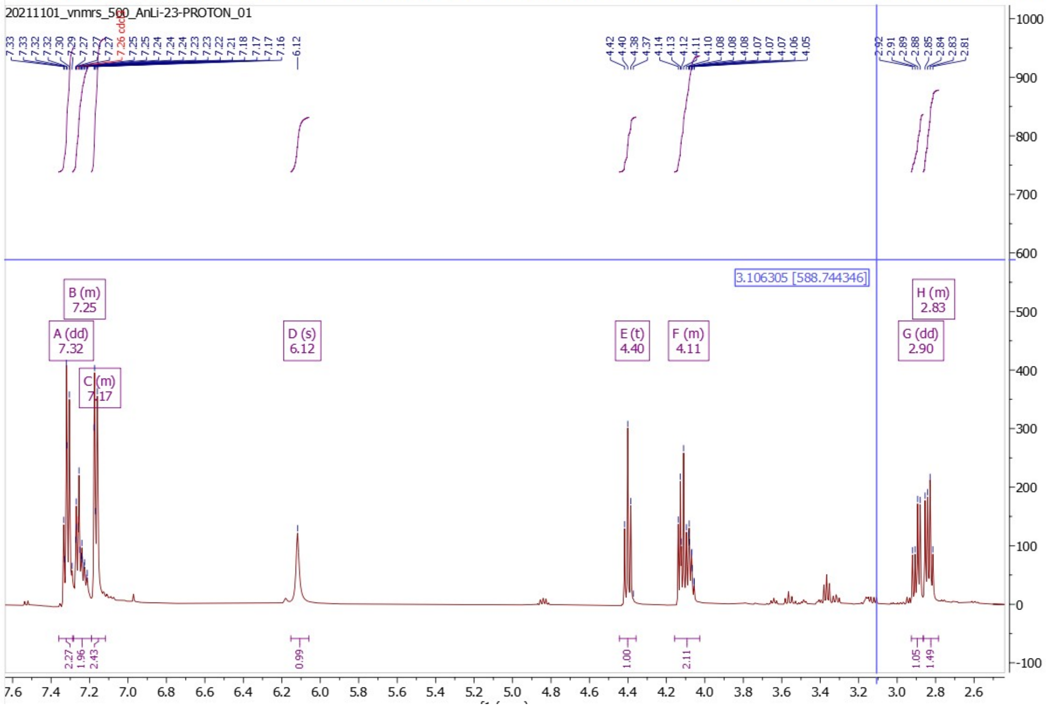

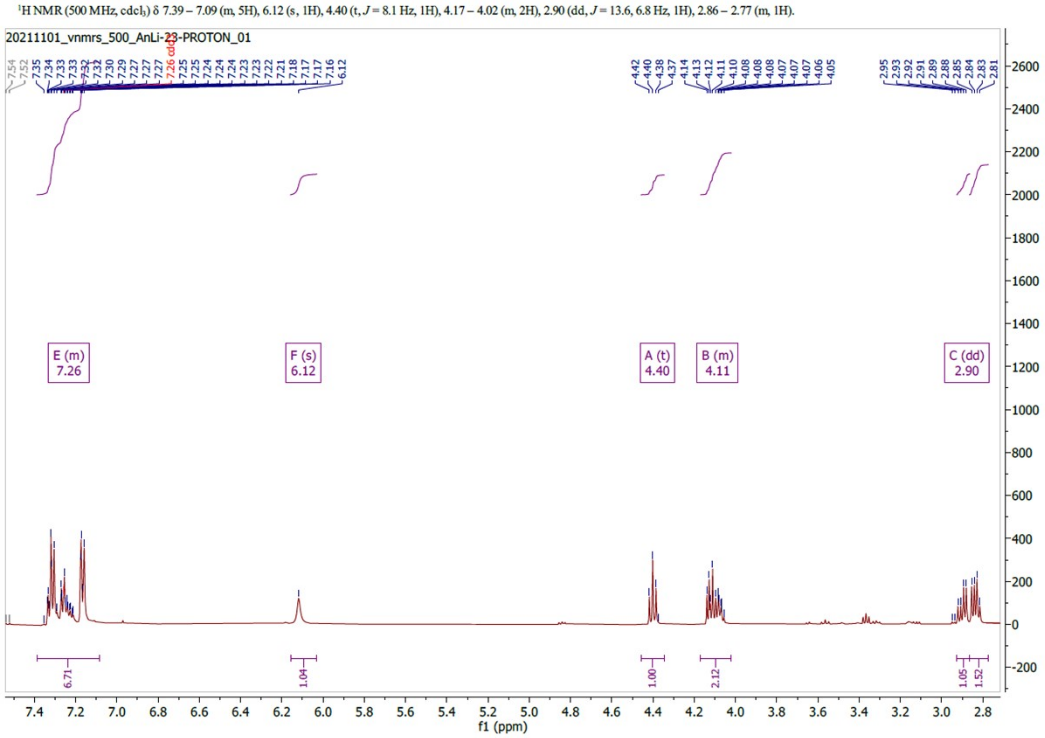

Figure S15. ^{1}H-NMR of 4-benzyloxazolidin-2-one (**1**)

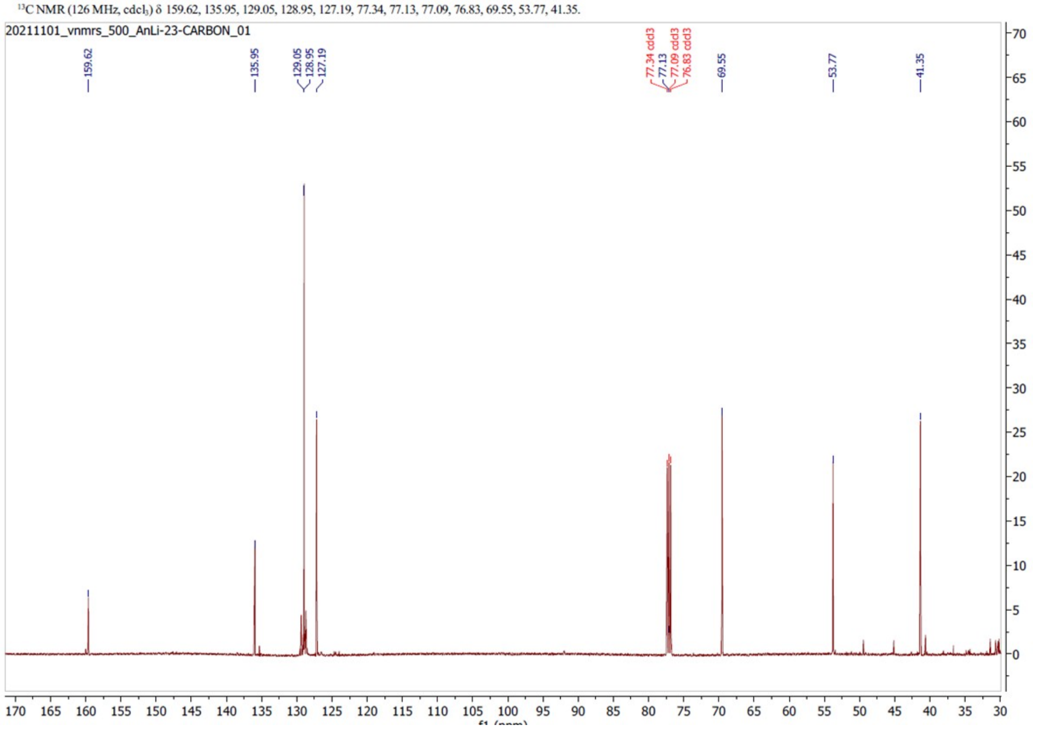

Figure S16. ^{13}C-NMR of 4-benzyloxazolidin-2-one (**1**)

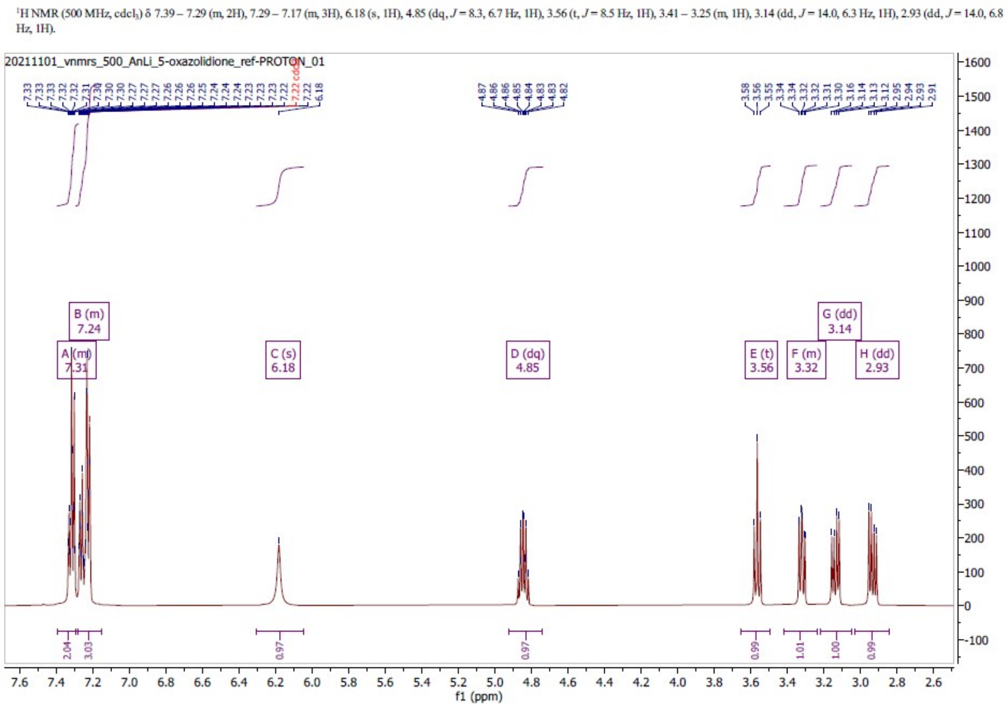

Figure S17. ¹H-NMR of 5-benzyloxazolidin-2-one (reference). ¹H NMR (500 MHz, $cdcl_3$) δ 7.39 – 7.29 (m, 2H), 7.29 – 7.17 (m, 3H), 6.18 (s, 1H), 4.85 (dq, *J* = 8.3, 6.7 Hz, 1H), 3.56 (t, *J* = 8.5 Hz, 1H), 3.41 – 3.25 (m, 1H), 3.14 (dd, *J* = 14.0, 6.3 Hz, 1H), 2.93 (dd, *J* = 14.0, 6.8 Hz, 1H).

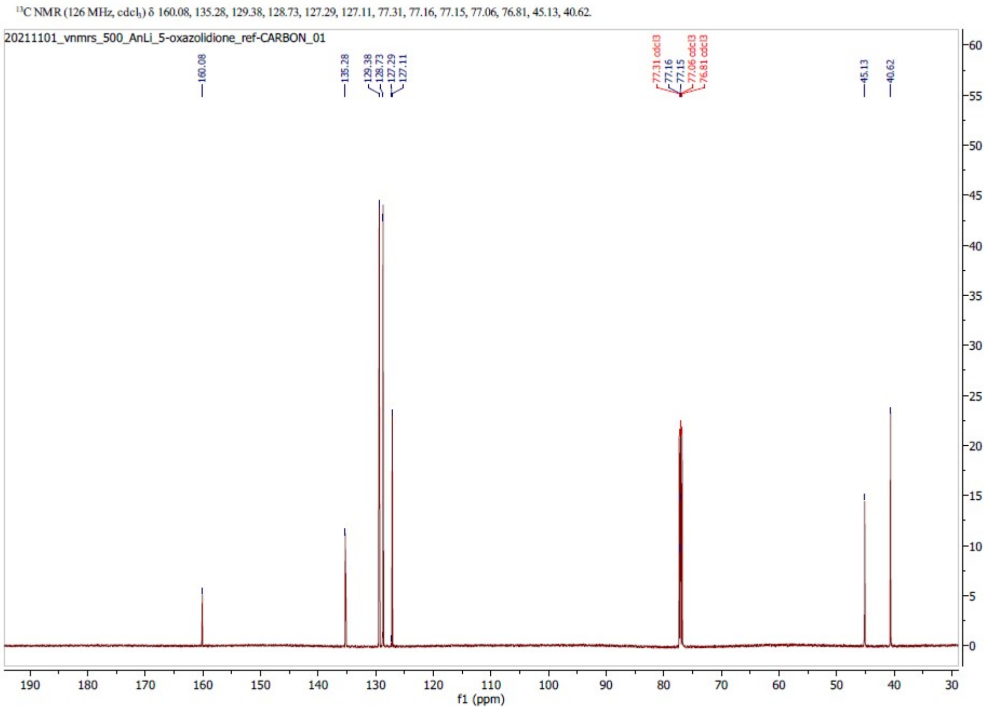

Figure S18. ¹³C-NMR of 5-benzyloxazolidin-2-one (reference). ¹³C NMR (126 MHz, $cdcl_3$) δ 160.08, 135.28, 129.38, 128.73, 127.29, 127.11, 77.31, 77.16, 77.15, 77.06, 76.81, 45.13, 40.62.

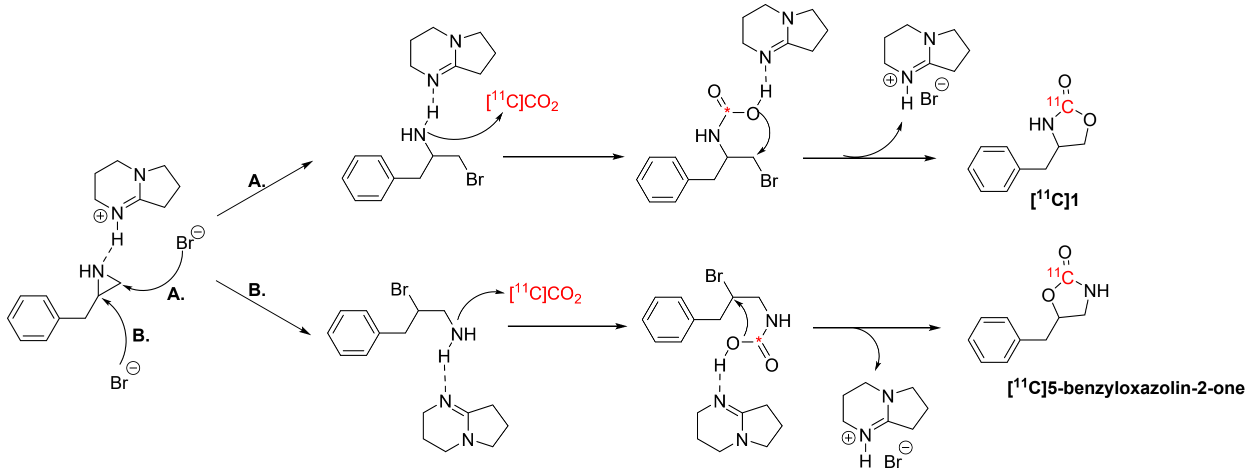

Scheme S1. Proposed mechanism for ring-opening of benzylaziridine with [11C]CO_2 with HBNBr. **A.** Mechanism resulting in [11C]4-benzyloxazolidin-one (**[11C]1**). **B.** Mechanism resulting in [11C]5-benzyloxazolisin-2-one.
